# Slowdown of BCM plasticity with many synapses

**DOI:** 10.1007/s10827-019-00715-7

**Published:** 2019-04-05

**Authors:** Maxime Froc, Mark C. W. van Rossum

**Affiliations:** 1 ENSTA ParisTech, Paris, France; 2 School of Psychology and School of Mathematical Sciences, University of Nottingham, Nottingham, NH7 2RD UK

**Keywords:** Synaptic plasticity, BCM, Dynamical systems

## Abstract

During neural development sensory stimulation induces long-term changes in the receptive field of the neurons that encode the stimuli. The Bienenstock-Cooper-Munro (BCM) model was introduced to model and analyze this process computationally, and it remains one of the major models of unsupervised plasticity to this day. Here we show that for some stimulus types, the convergence of the synaptic weights under the BCM rule slows down exponentially as the number of synapses per neuron increases. We present a mathematical analysis of the slowdown that shows also how the slowdown can be avoided.

Unsupervised learning describes how neurons change their responses when exposed to stimulation, even in the absence of any reward or teaching signal. This process is biologically particularly important during development of, for instance, the visual cortex and is believed to lead to the emergence of receptive fields well suited for further processing (Hyvärinen et al. 2009). The changes in the neural responses appear mostly mediated through long-term synaptic plasticity. A number of computational models has been introduced to describe unsupervised learning. One of the earliest models that gave a decent description of experimental data is the so called Bienenstock-Cooper-Munro model (Bienenstock et al. 1982; Cooper and Bear 2012; Cooper et al. 2004).

This note comes from an observation we made when simulating the BCM plasticity rule for a single neuron. We found that convergence could take very long, in particular when we increased the number of synapses modeled. We stimulate a neuron with 8 to 18 synapses with von Mises stimuli, centered randomly around any of the inputs (see *Simulation setup* for details), Fig. 1a. The weights are initialized close to a stable fixed point at which the neuron will be selective to one input only. We plot the angle between the weight vector and its final value as a function of time. The learning becomes exponentially slower when the neuron has more synapses, Fig. 1b (top). The number of synapses used is quite small. As biological neurons have thousand of synapses, this effect can seriously complicate simulation of neurons with a realistic number of synapses. In the bottom panel, we repeat the same simulation for triangular shaped stimuli. This minor modification speeds up the learning many orders of magnitude and the exponential slowing down is strongly reduced, Fig. 1b (bottom).

**Fig. 1:**
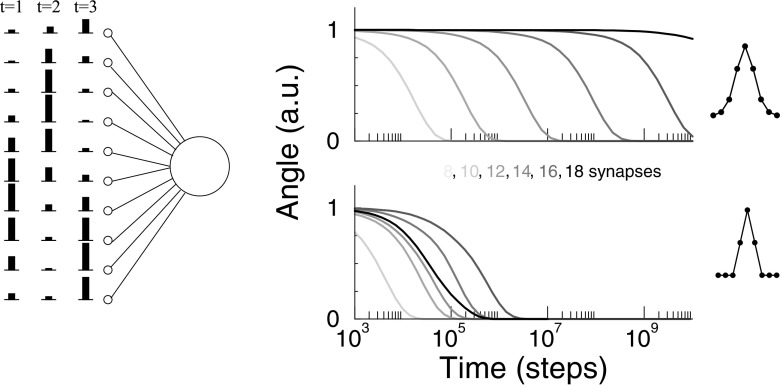
Slowdown of BCM learning for certain stimuli as the number of synapses is increased. **a**) Setup: Each time step the single neuron is stimulated with a stimulus with its peak at a random location (wrap-around boundaries). Synapses are modified according to the BCM rule. **b**) Top: When smooth stimuli are used, the convergence of the learning slows down exponentially as the number of synapses is increased from 8 (light) to 18 (dark). Bottom: There is no consistent slowdown when triangular-shaped stimuli are used. Convergence was measured as the angle between the weight vector and the weight vector in the steady state (normalized for visualization). See *Simulation setup* for other details

## The BCM model

We consider the standard BCM plasticity model for a neuron with *N* synapses

1 $$ \begin{array}{@{}rcl@{}} \frac{dw_{i}(t)}{dt} & =& F_{i}(\boldsymbol{w},\boldsymbol{x}) \end{array} $$

2 $$ \begin{array}{@{}rcl@{}} & =& \frac{1}{\tau_{w}}x_{i}y(y-\theta) \end{array} $$

where ***w***(*t*) is the synaptic weight vector with components *w*_*i*_(*t*) and *i* = 1…*N*. For mathematical convenience we assume *N* to be even. The input strength at input *i* is written *x*_*i*_. The neuron’s output rate is denoted *y* and here we use a linear relation between input and output $y={\sum }_{i}w_{i}x_{i}$. Essential to BCM, the threshold *𝜃*(*t*) tracks the average post-synaptic activity squared with a time-constant *τ*_*𝜃*_

3 $$ \tau_{\theta}\frac{d\theta(t)}{dt}=-\theta(t)+y^{2} $$

Together, Eqs. (1) and (3) form a (*N* + 1)-dimensional dynamical system.

We consider the commonly studied case that the inputs are sampled from a discrete distribution. In particular we initially assume that the inputs randomly alternate between *K* = *N* stimuli. One well-studied question is then: What are the values that the weights attain after learning has converged and equilibrated?

We write a specific stimulus vector as ***x***^*k*^, where index *k* = 1…*K* indexes the stimulus. The equilibrium condition for learning becomes that for all synapses, i.e. all *i*,

4 $$ {\sum}_{k}{x_{i}^{k}}y^{k}(y^{k}-\theta)= 0 $$

where *y*^*k*^ is the neural response to stimulus *k*. We will assume that the parameters are taken such that the system converges to a fixed point, which means that the learning rate (1/*τ*_*w*_) should be slow, and the threshold update rate (1/*τ*_*𝜃*_) should be relatively fast (Udeigwe et al. 2017). In that case we can replace the threshold by the average over the stimuli $\theta =(1/K){\sum }_{k}(y^{k})^{2}$. In other words, the dynamical system becomes *N*-dimensional. As an aside we note that the eigenvalue analysis presented below can also be done for the full *N* + 1-dimensional system (***w***,*𝜃*); in the limit of *τ*_*w*_ ≫ *τ*_*𝜃*_ this gives numerically identical results.

Next, we assume that all stimuli are identical but they are randomly centered at any of the synapses, Fig. 1a. This is in rough analogy with the visual system where the neurons are subject to stimuli with many different orientation, but during development become selective to a particular orientation. Furthermore we assume that the individual stimuli are symmetric around their peak response *f*_0_, so that ***x***^*k*^ = (…,*f*_2_,*f*_1_,*f*_0_,*f*_1_,*f*_2_,…). We introduce the matrix *X* that summarizes all stimuli so that ${x_{i}^{k}}=X_{ik}$

$$ X=\left( \begin{array}{ccccc} f_{0} & f_{1} & f_{2} & {\ldots} & f_{1}\\ f_{1} & f_{0} & f_{1} & {\ldots} & f_{2}\\ f_{2} & f_{1} & f_{0} & {\ldots} & f_{3}\\ {\vdots} & {\vdots} & {\vdots} & {\ddots} & \vdots\\ f_{1} & f_{2} & f_{3} & {\ldots} & f_{0} \end{array}\right) $$

Provided that the ***x***^*k*^ span a complete basis (i.e. det(*X*)≠ 0), the only solution to the equilibrium equations, Eq. (4), is that for every stimulus either *y*^*k*^ = 0 or *y*^*k*^ = *𝜃*. These conditions correspond to the fixed points in the learning dynamics.

It is known that the only *stable* fixed points are those where the neuron becomes selective to one single input pattern and remains silent for all other input patterns; fixed points where the neuron is active for more than one input or no input at all, are unstable (Castellani et al. 1999). The corresponding weights at the fixed points can be found directly as columns of the inverse stimulus matrix, i.e. $w_{i}^{*}=N(X^{-1})_{ik}$, where the pre-factor *N* follows from the threshold.

## Approach to equilibrium

We study how quickly the dynamics approach the fixed points. We denote the fixed points in the space of synaptic weights by ***w***^***∗***^. Close to the fixed points we can linearize the dynamics of Eq. (1) as $\tau _{w}\frac {d\boldsymbol {w}(t)}{dt}=J.(\boldsymbol {w}(t)-\boldsymbol {w}^{*})$, where Jacobian matrix is

$$ \begin{array}{@{}rcl@{}} J_{ij} & = &\frac{\partial F_{i}}{\partial w_{j}}|_{w=w^{*}}\\ & = & -N\sum\limits_{k = 1}^{K}{x_{i}^{k}}{x_{j}^{k}}\\ & = &-NXX \end{array} $$

Like the matrix *X*, the Jacobian matrix *J* is symmetric (*J*_*ij*_ = *J*_*ji*_) and circulant (in a circulant matrix each row is rotated one position w.r.t. the previous one). The eigenvalues of circulant matrices are identical to the Fourier coefficients of a row vector. Because *f* is symmetric, we can write it as a cosine series. Thus the eigenvalues of matrix *X* are the Fourier coefficients $a_{m}={\sum }_{j = 0}^{N-1}f_{j}\cos \left (2\pi j m/N\right )$, with *f*_*N*−*j*_ = *f*_*j*_, and *m* = −*N*/2 + 1,…,*N*/2. Hence the eigenvalues of the matrix *J* are $-N{a_{m}^{2}}$.

For the von Mises function, the eigenvalue with *m* = *N*/2 is closest to zero. Thus the most rapidly fluctuating spatial Fourier mode determines the convergence speed of the learning. It is given by the alternating sum

5 $$ \begin{array}{@{}rcl@{}} \lambda_{\text{crit}} &= & -Na_{N/2}^{2} \end{array} $$

6 $$ \begin{array}{@{}rcl@{}} &= & -N\!\left( f_{0} - 2f_{1}+ 2f_{2} - \ldots\pm2f_{N/2-1}\mp f_{N/2}\right)^{2} \end{array} $$

from which the slowest time-constant follows as *τ*_crit_ = *τ*_*w*_/*λ*_crit_.

**Fig. 2:**
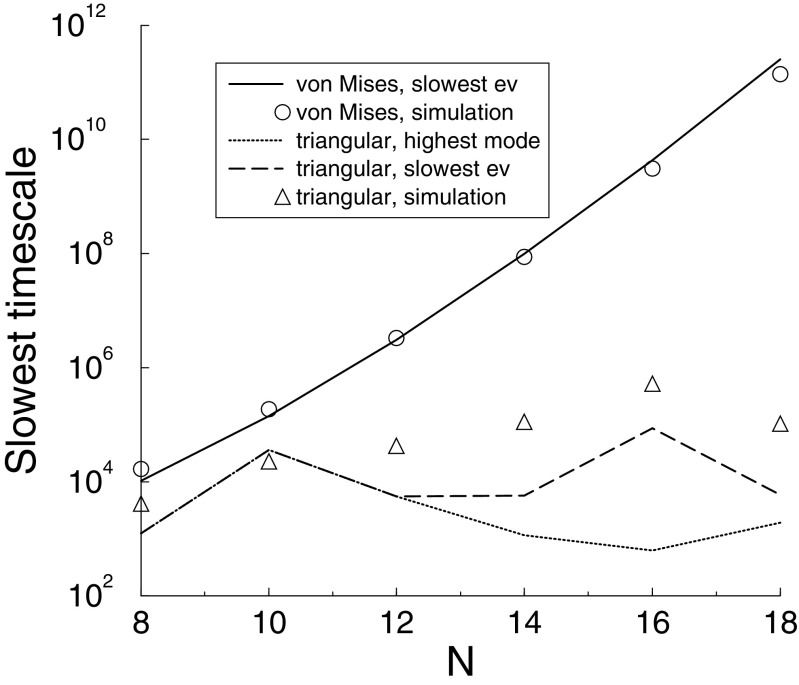
The slowest time-scale (the inverse of the smallest eigenvalue) for various stimuli. For von Mises stimuli (solid line), the time scale increases exponentially with the number of inputs. This matches the simulation results extracted from Fig. 1 (circles). For triangular stimuli there is no such increase. Furthermore, for the triangular stimuli the eigenvalue corresponding to the highest Fourier-mode (dotted) is not necessarily the slowest eigenvalue (dashed). The simulations reflect this as well (triangles)

What happens when the number of synapses *N* is increased? From Eq. (5) one would expect the magnitude of the eigenvalue to increase as *N*, and thus convergence would be quicker. However, if the stimulus is sampled from a smooth underlying function, the Fourier coefficient *a*_*N*/2_ will decrease as *N* increases, so that the time-constant slows down. In the case of von Mises stimuli the time-constant increases exponentially, Fig. 2. It is this effect that underlies the slowdown observed in Fig. 1. Indeed, the simulations (circles) match the theory (solid line), Fig. 2.

Another way to see that the smoothness of the underlying function matters, is to realize that the critical eigenvalue, Eq. (5), can be rewritten as the sum of discrete second derivatives of the stimulus $\lambda _{\text {crit}}\propto \left (f^{\prime \prime }_{1}+f^{\prime \prime }_{3}+f^{\prime \prime }_{5}+\ldots \right )^{2}$, where $f^{\prime \prime }_{i}=f_{i-1}-2f_{i}+f_{i + 1}$. The smaller this sum, the slower the convergence. This correctly predicts for instance, that increasing the width *ω* slows down the dynamics (not shown).

For triangular stimuli there is much less slowdown, Fig. 1 bottom. In this case the highest Fourier mode does not decrease with *N*. For non-smooth stimuli, highest Fourier mode is also not necessarily the critical eigenvalue, Fig. 2. Finally, the simulation do not match particularly well (triangles), as likely many modes contribute to the convergence.

**Fig. 3:**
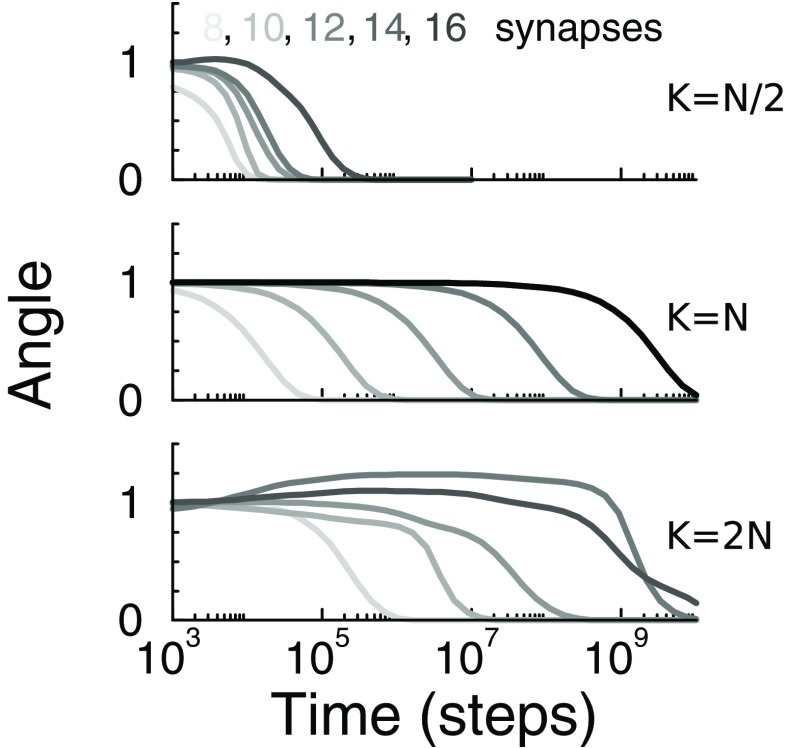
Convergence for under-complete and over-complete stimulus ensembles. The stimuli were *N*/2, *N*, or 2*N* equi-spaced von Mises profiles. Convergence speed was slow when the number of stimuli was equal or larger than the number of synapses. Note, for the cases where *K*≠*N* the initial weight vector was a random vector, resulting in a more random trajectory

Finally, we wondered if the finding was specific for the case where the number of stimuli equals the number of inputs (*K* = *N*). While we have not been able to extend the theory to this case, the situation can be simulated easily, Fig. 3. We find that when *K* = *N*/2, the convergence speed no longer strongly depends on the number of synapses, but for *K* = 2*N*, the slowdown is still present.

## Discussion

In short we have demonstrated both in simulation and by analysis that for some stimulus profiles BCM plasticity converges exponentially more slowly as the number synapses is increased. The reason is that the dynamics is proportional to the magnitude of the highest spatial frequency in the stimulus. As one increases the number of synapses and samples the stimulus more finely, this magnitude decreases for smooth stimuli.

While this turns out to be an important factor in the convergence speed of BCM learning, we stress that the analysis is by no means a full quantitative theory. The mathematical analysis presented here obviously required quite a few assumptions and approximations. The convergence speed will in general also depend on the initial conditions, and the other eigenvalues. Furthermore, it would be of interest to know how the convergence is affected by noise, heterogeneity, and how convergence proceeds far away from the equilibrium.

The slowdown is certainly relevant for researchers simulating the BCM rule. Whether these findings also carry a prediction for biology, that is, whether a similar slow down could be observed experimentally, will however require more research.

## Simulation setup

We simulated the BCM learning rule using as input ${x_{i}^{k}}=f_{|i-k|}=\exp \{[\cos (\frac {2\pi }{N}(i-k))-1]/\omega \}$, with width *ω* = 1/2. Von Mises functions are smooth periodic functions similar to Gaussian functions. Triangular stimuli defined as *f*_*i*_ = max(1 −|*i*|/(*ωN*),0), with *ω* = 0.38. The stimuli were repeatedly presented in a randomly permuted order. The *τ*_*w*_ was set to 1000 steps.

The initial weights were set as a unequal mix of two fixed points, ***w***(*t* = 0) = (1 − *𝜖*)***w***^1∗^ + *𝜖****w***^2∗^, with *𝜖* = 0.1 and ***w***^*k*∗^ = *NX*^− 1^***e***^*k*^, so the dynamics was prone to converge to ***w***(*t* →*∞*) = ***w***^1∗^. C and Octave code is available from the corresponding author.
